# Investigation of sensory attenuation in the somatosensory domain using EEG in a novel virtual reality paradigm

**DOI:** 10.1038/s41598-025-87244-9

**Published:** 2025-01-22

**Authors:** Gianluigi Giannini, Till Nierhaus, Felix Blankenburg

**Affiliations:** 1 Neurocomputation and Neuroimaging Unit (NNU), Freie Universität Berlin, Berlin, Germany; 2 Berlin School of Mind and Brain, Humboldt Universität zu Berlin, Berlin, Germany

**Keywords:** Cognitive neuroscience, Sensorimotor processing, Somatosensory system, Human behaviour

## Abstract

We are not only passively immersed in a sensorial world, but we are active agents that directly produce stimulations. Understanding what is unique about sensory consequences can give valuable insight into the action-perception-cycle. Sensory attenuation is the phenomenon that self-produced stimulations are perceived as less intense compared to externally-generated ones. Studying this phenomenon, however, requires considering a plethora of factors that could otherwise interfere with its interpretation, such as differences in stimulus properties, attentional resources, or temporal predictability. We therefore developed a novel Virtual Reality (VR) setup which allows control over several of these confounding factors. Furthermore, we modulated the expectation of receiving a somatosensory stimulation across self-production and passive perception through a simple probabilistic learning task, allowing us to test to what extent the electrophysiological correlates of sensory attenuation are impacted by stimulus expectation. Therefore, the aim of the present study was twofold: first we aimed validating a novel VR paradigm during electroencephalography (EEG) recoding to investigate sensory attenuation in a highly controlled setup; second, we tested whether electrophysiological differences between self- and externally-generated sensations could be better explained by stimulus predictability factors, corroborating the validity of sensory attenuation. Results of 26 participants indicate that early (P100), mid-latency (P200) and later negative contralateral potentials were significantly attenuated by self-generated sensations, independent of the stimulus expectation. Moreover, a component around 200 ms post-stimulus at frontal sites was found to be enhanced for self-produced stimuli. The P300 was influenced by stimulus expectation, regardless of whether the stimulation was actively produced or passively attended. Together, our results demonstrate that VR opens up new possibilities to study sensory attenuation in more ecological valid yet well-controlled paradigms, and that sensory attenuation is not significantly modulated by stimulus predictability, suggesting that sensory attenuation relies on motor-specific predictions about their sensory outcomes. This not only supports the phenomenon of sensory attenuation, but is also consistent with previous research and the concept that action actually plays a crucial role in perception.

**Supplementary Information:**

The online version contains supplementary material available at 10.1038/s41598-025-87244-9.

## Introduction

Our sensorium appears to use internal models to predict future sensory data and thus also to filter environmental noise from salient information, so that our cognitive capacities can be allocated to optimally process information that is innovative or useful^1–4^. We are, however, not only passively immersed in a perceptual world. Every time we move or directly act on our environment, we generate a sequence of sensory data that can be predicted with a higher precision than external data^5^. This might be responsible, amongst others, for our ability to perceive ourselves as self-standing agents^6,7^ or for distinguishing our speech from that produced by others^8,9^. These concepts also nicely fits within the framework of active sensing^10–12^, in which actions play a proactive role in shaping sensory experience, wherein movements are not just passive receivers of sensory input but actively generate predictions about the sensory consequences of those movements to gather information. The minimisation of the interference from predictable signals, has also been argued to result in authorship attribution or – what is commonly defined – sense of agency^13^ and to be disrupted in schizophrenia^14–16^, indicating a possible link between predictive mechanisms and selfhood^17^.

Among the multiple phenomena emerging from this interaction between action and perception, sensory attenuation is the phenomenon that self-generated stimulations are suppressed compared to similar externally-generated stimuli^18,19^, both at the subjective^20–26^ and neurophysiological level^19,27–34^. It is suggested that at the core of sensory attenuation lies a forward model: upon the generation of a motor command, an efference copy is evoked to predict the sensory consequences of that movement^35^. These motor predictions and their observed sensory consequences are then compared. If the prediction matches the sensory re-afference, the self-generated stimulation is attenuated or cancelled-out and therefore perceived as less intense^18,21,36^.

Historically, the investigation of the electrophysiological mechanisms underlying sensory attenuation has been largely studied using so-called contingent paradigms^37^. In these setups, participants undergo three conditions which require them to (i) perform sequences of prompted actions with a contingent sensory consequence, (ii) passively undergo similar sequences of stimuli generated by a computer and (iii) perform sequences of actions without any sensory consequence. Typically, the *motor-only* condition is subtracted from the *motor-and-sensory* condition to obtain a motor-corrected potential of self-generated stimulation that is then compared to the *sensory-only* potential of passively perceived stimuli. Electroencephalography (EEG) typically indicates that event-related potentials (ERPs) at around 100 ms and 200 ms post stimulus are attenuated for self- compared to externally-generated stimulations, either in the auditory^16,19,38–41^, visual^42–46^, and, although more scarce in number, in the somatosensory domain^47,48^.

Despite the extensive work that has been published in recent years, the investigation of sensory attenuation has often revealed to be difficult in controlling for other explanatory factors. First of all, most studies investigating the electrophysiological correlates of sensory attenuation required participants to execute button presses to generate sensory action consequences^16,19,43,44,49,50^. It has been argued, however, that the additional mechanical stimulation from physically pressing a button – which is entirely perceived during *motor-only* conditions – would be masked by the self-generated sensory stimuli in the *motor-and-sensory* condition. The later subtraction of the two ERPs would then result in a smaller component, thus mimicking an attenuation for self-produced stimuli^37,51^. Moreover, albeit experimental stimulations in sensory attenuation paradigms are kept constant, additional mechanical stimulations exerted upon button press would be dependent on the velocity of the participants’ movements or the applied force and might therefore remain uncontrolled. Also, task differences in the classical contingent paradigm (i.e., passive listening compared to actively pressing a button to generate a sound) might induce an imbalance in attention requirements across conditions, which could account for the suppression effect reported between self- and externally-generated stimuli. In fact, as it is well known that attention requirements might reduce the electrophysiological responses evoked by a stimulation^52–58^, it has been discussed that the simple execution of a motor task might require to allocate attentional resources away from stimulus perception, which might determine an ERP suppression for self-generated stimulations^37,59^. Another component that might affect the investigation of sensory attenuation is stimulus predictability^60^. Previous studies that manipulated stimuli predictions at rest suggested that stimuli that are predictable either in time^19,61–63^, in the identity of the stimulation^64–69^ or concerning the expectation of receiving a stimulus^70,71^ exert an evoked electrophysiological response that is attenuated compared to the relative unpredictable counterpart. Studies that tried to control for these factors typically report that suppression of self-generated stimuli is resilient to temporal predictability^31,50^ or stimulus identity predictions^22,28,49,72^, despite some findings reporting mixed evidence^73^ or proving the contrary^29^. This evidence not only points out the necessity of exerting a broader control over different factors that might confound the interpretation of electrophysiological attenuations for self-generated stimuli, but also puts at risk the concept of sensory attenuation itself: it is becoming increasingly difficult to trace a clear line whether sensory attenuation indeed relies on internally generated and motor specific predictions or rather unspecific ones.

In order to address these issues, new methodological instruments might be useful. In particular, Virtual Reality (VR) and head mounted displays are capable of creating immersive settings with a high degree of freedom^74^ and are especially interesting for the exploration of action and perception interrelations. They allow the creation of setups in which different sensory and motor components are manipulated at will in ways that would not be feasible in other experimental settings. Only a handful of studies adopted such technologies in the investigation of sensory attenuation^75–78^ and only one research integrated a VR setup with an EEG recording^79^.

The current study expands on this work by collecting electrophysiological responses to self-generated and passively attended stimuli in a VR setting. The paradigm was designed to compare the electrophysiological potentials evoked by an electrical pulse at the fingertip resulting from a goal-directed action with the same potential when passively attended, minus of a no-stimulation control condition (similar to a contingent paradigm). Also, because the stimulations were administered in a probabilistic fashion either when self-produced or passively attended, we could also test for the influence of stimulus predictability on sensory attenuation. We hypothesized that modifications of (EEG) activity evoked by the stimulation at early and mid-latencies (typical findings of sensory attenuation) would be explained by either self-producing or passively attending the stimulation, suggesting that sensory attenuation is based indeed on motor-specific predictions. On the contrary, if differences in the EEG activity would be better explained by stimulus predictability, then this would indicate that sensory attenuation is relying on unspecific identity prediction mechanisms. Nonetheless, stimulus predictability was expected to modulate the later P300 component, independent of whether the stimulation was self- or externally produced. This would substantiate the concept that actions indeed shape the perception of their resulting sensory consequences, possibly aiding other higher cognitive functions such as authorship attributions and selfhood perception.

## Materials and methods

The task consisted of a probabilistic learning paradigm in VR and concurrent EEG recording. The participants saw a 3-dimensional space though a VR headset in which they either actively reaching for or passively being touched (*move* or *stay* conditions) by a virtual ball that could give them an electrical stimulation (*touch* and *no-touch*) in a probabilistic manner (three levels of probability: *low*, *equal*, *high*). This paradigm allowed us to directly compare the electrophysiological correlates evoked by a stimulus in both active and passive conditions, while controlling for the subjective expectation of receiving the stimulation.

### Participants

26 healthy volunteers (18–35 years old, mean: 24.36, 16 females, all right-handed), recruited from the student body of the Freie Universität Berlin and the general public, participated for monetary compensation or an equivalent in course credit. The sample size was based on previous studies investigating sensory attenuation using a similar design (Kaiser et al., 2018; Bednark et al., 2015). The study was approved by the ethics committee at the Freie Universität Berlin (003/2021), and it was performed in accordance with the declaration of Helsinki. Written informed consent was obtained from all participants prior to the experiment.

### Experimental setup / apparatus

**Fig. 1:**
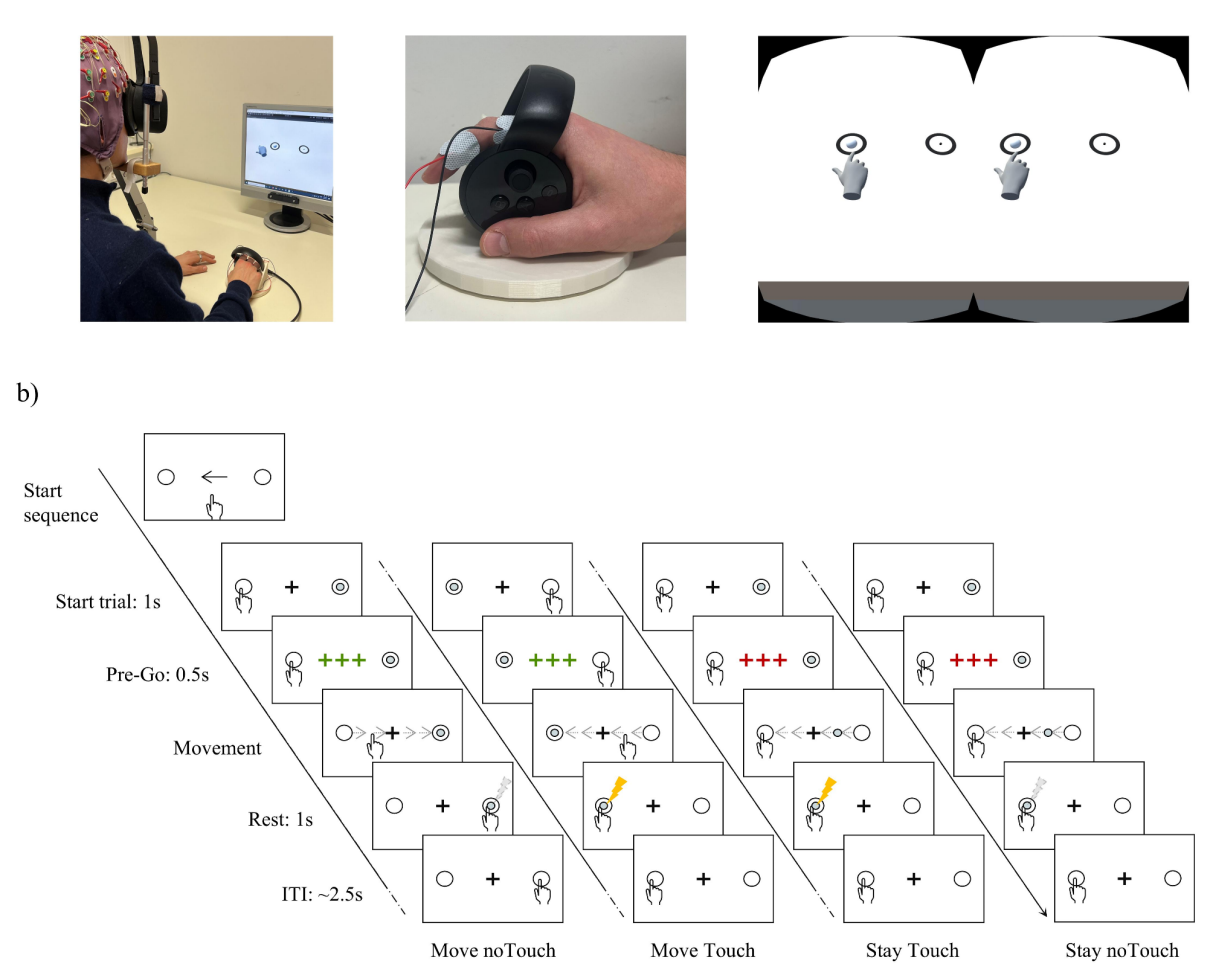
Experimental setup. (**a**) On the left, Oculus headset mounted on top of a chinrest, in which the participant puts his head while wearing the EEG cap. The volunteer is holding the oculus controller mounted on the 3D sliding support. On the computer monitor, Unity is displaying a replica of the scene rendered 3D in the VR-headset. In the middle, a participant holding on the oculus controller. On the index finger, typical electrode positioning that was used throughout the experiment for administration of electrical stimuli. On the right, rendered scene that was visible to each eye. (**b**) Depiction of an example of a sequence of trials in the experimental paradigm. At the start of the sequence, the participant saw at the centre of the screen an arrow indicating where to position their index finger (left-most frame). Once the indicated position was reached, the trial sequence could start. On the first trial of the new sequence, the participant was instructed to reach and touch the virtual ball positioned in the opposite indicator circle (first column), which administered no electrical stimulation (grey flash). After reaching the new position (right indicator circle), the participant was required to stay still. The next trial (second column) started with the appearance of the virtual ball in the indicator circle opposite to the hand. The participant was instructed to move again and touch the ball, which resulted in an electrical pulse at the fingertip (yellow flash) and the reach of a new position (left indicator circle). In the following trials (third and fourth columns), the participant was requested to stay still and passively wait for the ball to reach their index finger. Starting from the first column, the following trials were represented in order: *move no-touch*, *move touch*, *stay touch*, *stay no-touch*.

The paradigm was presented in virtual reality (VR) using an Oculus Rift CV1 headset (Meta, Menlo Park, California, USA) mounted on top of a chinrest. This setup allowed to minimise electrical and mechanical artifacts generated by wearing the headset directly on top of the EEG cap^80–82^. Somatosensory stimuli were administered with a DS7 isolated bipolar constant current stimulator (Digitimer Limited, Welwyn Garden City, Hertfordshire, UK) via adhesive electrodes (GVB-geliMED GmbH, Bad Segeberg, Germany) attached to the outer side of the right index finger (cathode proximal, anode distal, see Fig. 1a, left). The stimuli consisted of electrical rectangular pulses of 0.2 ms duration.

The VR scene was built using Unity v.2020.3.26f1 (Unity Technologies, San Francisco, California, USA). To mimic as accurately as possible the real-world setup, proportions of the virtual objects were scaled according to a ratio of 1 Unity units (Uu) to 1 real-world meter. A virtual white cube resembling a white table was positioned in a grey room. The camera from which participants could see the scene, was placed 0.35 Uu behind the table and 1.3 Uu from the ground, with a tilt of 66° degrees, resembling the same point of view as if participants were looking at their right hand moving on the table in the real world (see Fig. 1a, left). In the real world, participants were asked to hold an Oculus controller in the right hand, which was mounted on a 3d-printed sliding support created ad hoc (see Fig. 1a, centre). Throughout the experiment, participants controlled the movements across the horizontal plane (x and y) and rotations (across the z axis) of a virtual hand by moving the controller in the real world. The virtual hand was also rendered in a similar position to how participants were holding on the controller and its movements were locked on the z-axis on top of the table (no vertical movements were possible, just horizontal slides). Hand position and rotation along the three axes were recorded throughout the whole experiment with a time resolution of 86.30 Hz (SD = 0.45 Hz).

### Calibration and setup

At the beginning of a session, participant’s sensory threshold was determined by gradually increasing the stimulation intensity until they reported feeling the stimulation. Then, the amperage was modified until participants reported feeling 5 out of 10 pulses (mean: 1.96 mA, min: 1.20 mA, max: 3.40 mA). To ensure that each stimulation was clearly perceivable during the training and experimental sequence, participants received stimulation 1.5-2.0 x their subjective threshold, adjusted based on comfortability (mean: 3.63 mA, min: 2.33 mA, max: 6.80 mA). The same intensity was used throughout the experiment. Participants were also asked to report the position at which they felt the stimulation. Since they could have seen the virtual ball touching their fingertip either from the left or from the right side, we tried to ensure that the stimulation was perceived in the centre of the finger.

The height of the headset and the lenses focus were adjusted to obtain maximal visual resolution. Moreover, since participants were asked to perform leftwards and rightwards movements with their right hand (see later), we adjusted the correspondence between real-world and virtual-environment movement (i.e., calibration) so that movements in both directions were equally easy and comfortable. Then, the experimenter fitted the EEG cap to the participant and a short training phase of about 5 min started. The training phase consisted of 5 trials of the *move* condition, 5 trials of the *stay* condition and a full sequence of 25 trials. During the initial 10 sample trials, electrical stimulations were always administered to familiarise participants. During the full training sequence, stimulations were given in a probabilistic fashion, similarly as in the experimental phase. Lastly, during the training phase, we measured the velocity of participants’ movement and used it to adjust the velocity of the moving ball during *stay* conditions in the experimental phase. The average velocity and the variance across 14 *move* training trials (out of 25) was calculated. The virtual ball during *stay* trials moved at an average speed across participants of 0.41 Uu/s (SD = 0.08).

### Experimental design

In each of the 4 experimental runs of approx. 12 min, participants underwent 6 sequences of 25 trials, for a total of 600 trials per participant. A fixation cross appeared in the middle of the field of view of the camera, on the virtual table. To minimise horizontal eye movements, we instructed participants not to follow the moving ball or their hand but to keep their gaze on the cross. On the virtual table there were also two indicator circles (distance +/- 0.2 Uu from the fixation cross). Participants were instructed to either keep their index finger in the circle or move their finger towards the circle located in the opposite side of the virtual table, in the *stay* and *move* conditions respectively. Thus, movements will only appear between the two indicator circles. Both circles were within the field of view of each eye (see Fig. 1a, right) permitting participants to visualize the whole experimental setting without the necessity of performing eye movements (see also Fig. 1). Although participants were instructed to fixate a cross located in the centre of the table, sporadically, some eye movements occurred. During the experimental session, we monitored eye movements online by the electro-oculograms (EOG). Participants were informed about their eye movements between blocks when they ignored the instructions to fixate on the central cross, while trying to minimize eye movements.

At the beginning of each sequence of 25 trials, an arrow indicated the circle in which the participant had to put their index finger. Once the finger was in the circle, the new sequence started. Each trial began with the virtual ball appearing in the centre of the circle opposite from the participant’s finger. After a delay of 1 s, the fixation cross changed colour for 0.5 s. If the cross flashed green, participants were required to actively reach the ball positioned on the opposite side of the table (*move* condition). Participants were instructed to move as soon as the cross stopped flashing. If the cross flashed red, they were required to stay still and wait for the ball to reach their fingertip (*stay* condition). The ball started to move as soon as the cross stopped flashing. If participants moved during a *stay* condition, a prompt appeared indicating the wrong execution of the trial. Upon reaching and touching (or touched by) the virtual ball, an electrical shock at the fingertip could have been administered (*touch* and *no-touch* conditions). For a depiction of the experimental paradigm, see Fig. 1b. Electrical pulses were administered according to a simple probabilistic model with 25%, 50% and 75% probability (i.e., *low*, *equal*, *high*). This meant that even for less likely conditions (i.e., *touch move low probability*) there were in total approximately 25 trials, which is a similar requirement to other studies implying similar designs in the investigation of sensory attenuation^50^. Within each sequence, the probability of incurring in a *move* or *stay* trial was at chance level. Therefore, participants underwent the same number of *move* and *stay* trials and, most importantly, the likelihood of receiving a stimulation was not associated with the probability of moving or staying still. Participants were explicitly informed about this.

After subjects reached and touched (*move* condition) or were touched by (*stay* condition) the virtual ball, they were instructed to stay still and keep their index finger in the indicator point, waiting for a new trial. One second after touching the ball, the virtual object disappeared, and a new trial started after a random inter trial interval between 1.75 and 2.25 s. The next trial started always with the virtual ball appearing in the opposite indicator circle respect to the participant’s hand. Trial orders was pseudorandomised to account for the correct sequence of movements, i.e., when a participant concluded a trial with their finger in the left indicator circle, the next trial could either be a *stay* trial, with the ball coming from the right side towards the left or a *move* trial, instructing participants to move from the left position towards the right, where the ball was positioned.

At the end of each sequence of 25 trials, three circles with the labels “25%”, “50%”, and “75%” appeared on the screen. Each percentage was associated with a probability condition, namely *low*, *equal* or *high*. Participants had to slide their finger in the circle corresponding to the probability condition that they thought was the one underlying the stimulus presentation during the sequence. Participants’ task was therefore to learn the probability distribution underlying the administration of electrical pulses upon contact with the virtual ball and to report it at the end of each sequence.

### Behavioural data analysis

Probability discrimination accuracy rates were analysed only via descriptive statistics, due to scarcity of data-points. Given that we prompted participants to report the supposed underlying probabilistic state at the end of each sequence, we only had eight accuracy responses per probabilistic state.

Response times were calculated from the time of action (or ball moving) onset to the moment of ball touch (independent of electrical stimulation and for the *move* and *stay* condition, respectively). Outliers defined as trials exceeding 3 median absolute deviations were excluded. We fitted a linear mixed effect model having as fixed effects movement type (*move* or *stay*), stimulation (*touch* or *no-touch*) and probability condition (*low*, *equal*, *high*). Random intercepts were modelled by participants. This was done to check that our velocity personalisation approach across *stay* and *move* conditions was successful and that differences in trial lengths across conditions might have driven differences in the electrophysiological correlates.

### EEG data collection and preprocessing

Data were collected using a 64-channel active electrode EEG system (ActiveTwo, BioSemi, Amsterdam, Netherlands) at a sampling rate of 2048 Hz, with head electrodes placed in accordance with the extended 10–20 system.

Preprocessing of the EEG data was performed using SPM12^83^, FieldTrip^84^ and in-house MATLAB scripts. First, bad channels were identified manually and removed, the data were then referenced against the average reference, down-sampled to 512 Hz and high-pass filtered (0.01 Hz, firws, one-pass zero-phase, -6 dB cut-off). Subsequently, eye-blinks and horizontal eye-movements were corrected using a topographical confound approach^85,86^.

Next, we defined trials as the recording epochs going from at least half a second before instruction cue to at least one second after ball-touch. Due to the great variability in trial length in our experiment, this resulted in a trial definition that spanned from − 3s to 3s around ball-touch events. A low pass filter was applied (45 Hz, firws, one-pass zero-phase, -6 dB cut-off) and EEG data were baseline corrected with respect to the pre-stimulus interval from −50 to −5 ms. Finally, each trial was visually inspected and bad data segments were marked and excluded from the final dataset. To ensure that the data were equivalent between the EEG and the behavioural analyses, in both datasets we kept only trials that (i) did not contain any response time outlier and (ii) were artifact free. On average, we excluded a total of 12.32% of trials (SD = 8.34%). 1.29% (SD = 1.10%) of the total trials were response time outliers and 11.28% (SD = 8.16%) contained artefactual segments, while 0.25% were both. After exclusion, an average of 526 trials (SD = 50) survived. Moreover, out of the initial 26 participants, one was excluded because they never chose the 75% probability condition as a response. The results presented later are therefore computed on 25 participants.

### EEG data analysis

Main analyses were done within the SPM framework for M/EEG analysis. This method required the preprocessed, epoched channel data to be converted into a 3D image (scalp space x intra-trial samples). More specifically, each electrode was first laid within a 32 × 32 mask according to its position relative to a 2D projected standard 64 channel EEG cap, so that the position of the channels resembles the position of the sensors on the scalp. Then, the array of electrodes was linearly interpolated for each time-point to obtain a scalp similar distribution of the electrical potential over time for each epoch^87^. This procedure allowed to interpolate electrophysiological data arrays into 3D images to be further analysed with correction for multiple comparisons using random field theory^87,88^.

Because we were specifically interested in the electrophysiological responses evoked by the electrical stimulation, we selected a window spanning from − 50 to 500 ms around ball-touch. In this way, we obtained one 3-D image of dimensions 32 × 32 × 283 (scalp space x intra-trial samples) per trial. First-level multiple regression models were then specified and estimated in SPM12, using dummy regressors for each possible condition combination (12 in total). This allowed for the regression of the EEG data over trials, separately for each voxel, which resulted in a 3-D β estimate for each condition with the same dimensionality as the initial images. Each β estimate, without the inclusion of further regressors or covariates, was mathematically equivalent to computing the ERPs of each condition. Second level analyses consisted of a mass-univariate multiple regression analysis of the individual β scalp-time images with a design matrix having one regressor for each condition of interest as well as for each subject. Mean differences across conditions were tested via F-tests and therefore, in its interpretation our model is equivalent to a 2 × 2 × 3 ANOVA, having as factors: stimulation (*touch*, *no-touch*), movement (*move*, *stay*) and probability (*low*, *equal*, *high*). All analyses were performed with a cluster-forming threshold of *p* < 0.001; only clusters withstanding at the cluster-level with family-wise error (FWE) corrected threshold of *p*_FWE_ <0.05^89^ are reported here.

The direct comparison of potentials evoked by an electrical stimulation after a movement or while staying still will lead to a spurious difference, driven by the additive effect of the motor act in the *move* condition. In contingent paradigms, a *motor-only* condition is subtracted from a *motor-and-sensory* condition to obtain a corrected ERP of self-produced stimulus perception to be compared against a *sensory-only* condition. In a similar fashion, this problem was addressed in our design by selectively exploring the interaction term across conditions (i.e., stimulation x movement, stimulation x probability, stimulation x movement x probability). In this way, we could directly compare the potentials evoked by the self- and externally-produced electrical pulses, minus *move* or *stay* *no-touch* control conditions (similar approaches were also adopted in:^30^).

## Results

### Behavioural results

**Fig. 2:**
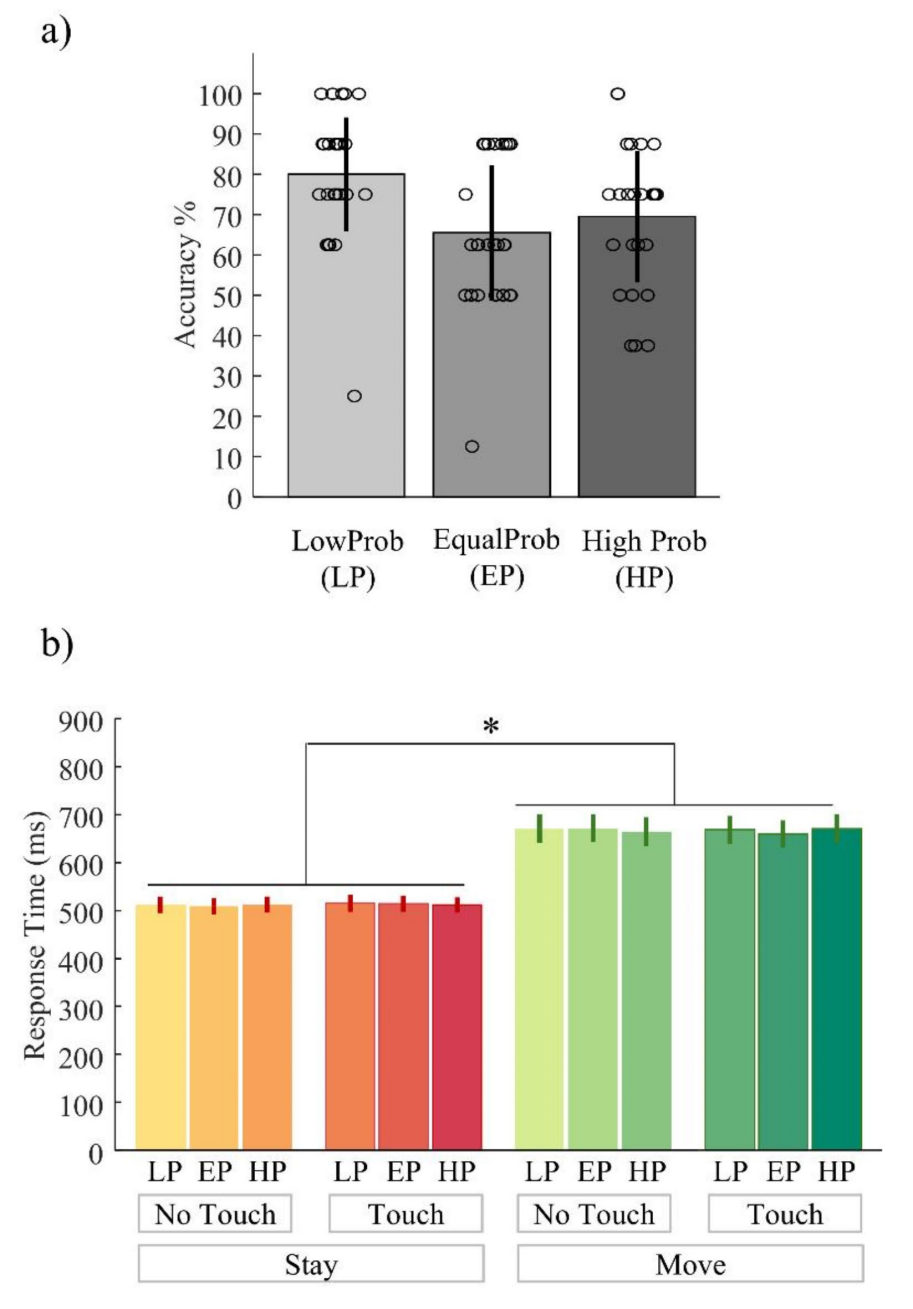
Behavioural results (**a**) Average accuracy scores obtained by all participants and categorized by probability condition. Error bars represent standard errors; circles are the condition-specific averages obtained by each participant. (**b**) Average response times for each condition. Error bars represent standard errors. The asterisks represent significant differences at *p* < 0.001. LP, EP, HP = *low*, *equal*, *high* probability, respectively.

Regarding accuracy measures, participants’ belief matched the real underlying state in 80% in the *low* condition, in the 65.5% of the *equal* and in the 69.5% of the *high* condition (see Fig. 2a). Participants’ performance was well above chance level (33.3%), i.e., the participants were able to perform the task.

We then fitted a linear mixed effect model to the response times to test for differences across condition. The model revealed a main effect of movement type (*F*_1,13104_ = 1646.9, *p* < 0.001) but no main effect of stimulation (*F*_1,13104_ = 0.008, *p* = 0.929) and probability condition (*F*_2,13104_ = 0.476, *p* = 0.621) or any interaction effect (see Fig. 2b).

### Electrophysiological results

#### Qualitative EEG description

**Fig. 3:**
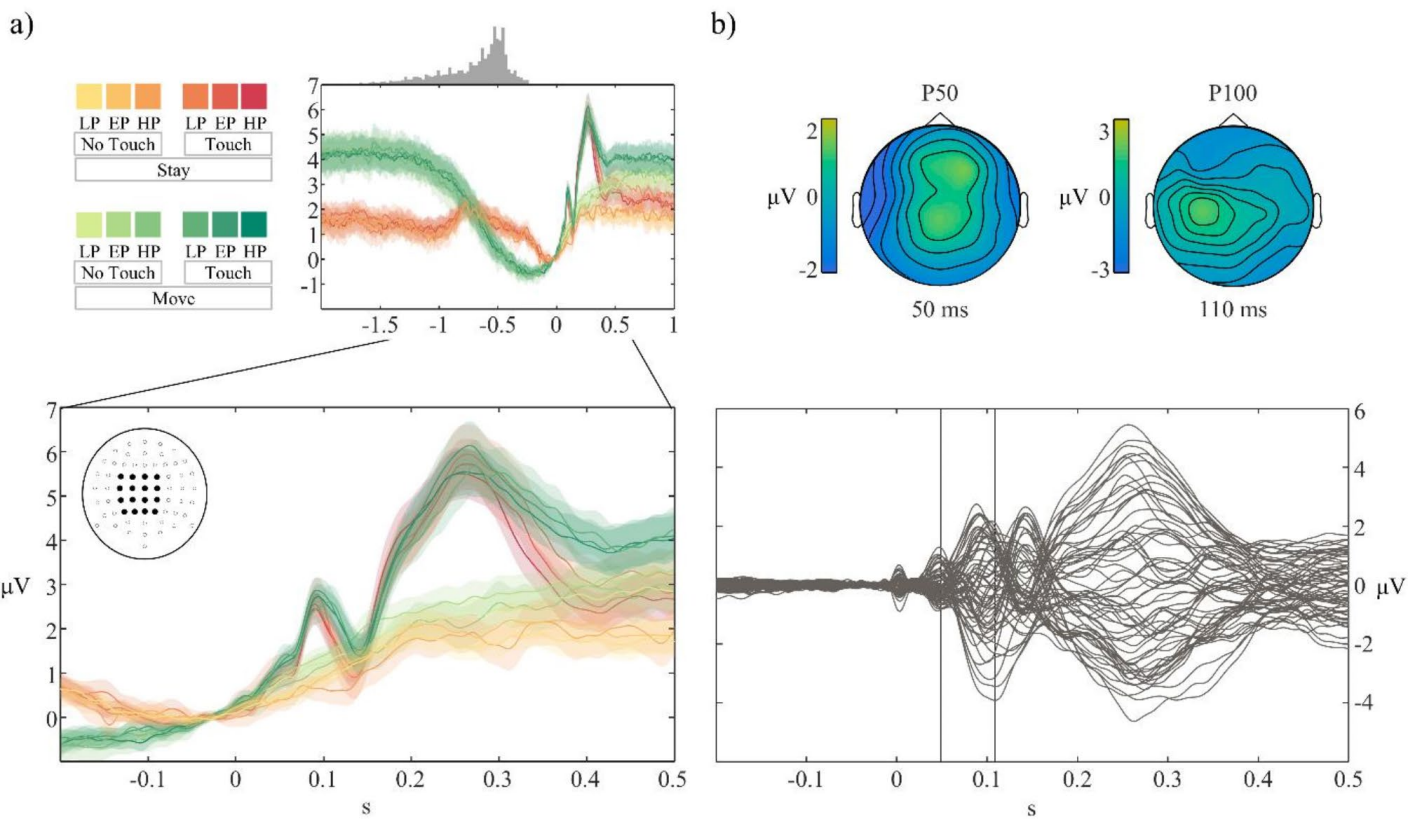
Qualitative analysis of potentials evoked by stimulus onset. (**a**) On the left, ERPs for each condition for a subset of centro-lateral electrodes (marked as darker dots in the head shaped topography in the upper left corner of the lower ERP plot). Juxtaposed on top of the ERP plot, histogram of movement onsets. On the bottom, detail of the ERPs for each condition for a subset of centro-lateral electrodes (marked in the head-shape plot in the upper left corner) in the time window that was later brought at the group level analysis. (**b**) Superimposed plot of all electrodes (butterfly plot) of the difference between the averaged *touch* trials subtracted of the averaged *no-touch* trials. On top, scalp distributions of the difference between *touch* and *no-touch* trials at 50 ms and 110 ms post-stimulus. LP, EP, HP = *low*, *equal*, *high* probability, respectively.

The averaged electrophysiological activity was characterised by an increasing pre-stimulus negativity in both the *stay* and *move* conditions that reached its peak at stimulus onset. The negativity observed in *stay* trials before stimulus administration most likely reflects a process of tactile stimulus anticipation^90–93^, while the negativity observed during *move* trials is due also to motor preparatory processes^94,95^ and slow frequencies related to motor execution^96–98^ (Fig. 3a, top). Thus, in the pre-stimulus period, time-locked electrophysiological activity differed between *move* and *stay* conditions. These differences were accounted for by the inclusion of the *no-touch* control condition.

From stimulus onset, our paradigm elicited the typical somatosensory responses^99,100^ (Fig. 3a, bottom). Figure 3b shows the difference between the average *touch* trials across participants subtracted of the *no-touch* trials across electrodes with the expected evoked potentials, i.e., P50, N140 and P300 resulting from electrical stimulation of the right index finger. The corresponding topographic maps (Fig. 3b, top) confirm the left lateralized voltage distribution of the somatosensory evoked potential (SEP) components on the scalp.

#### Quantitative EEG analysis

**Fig. 4:**
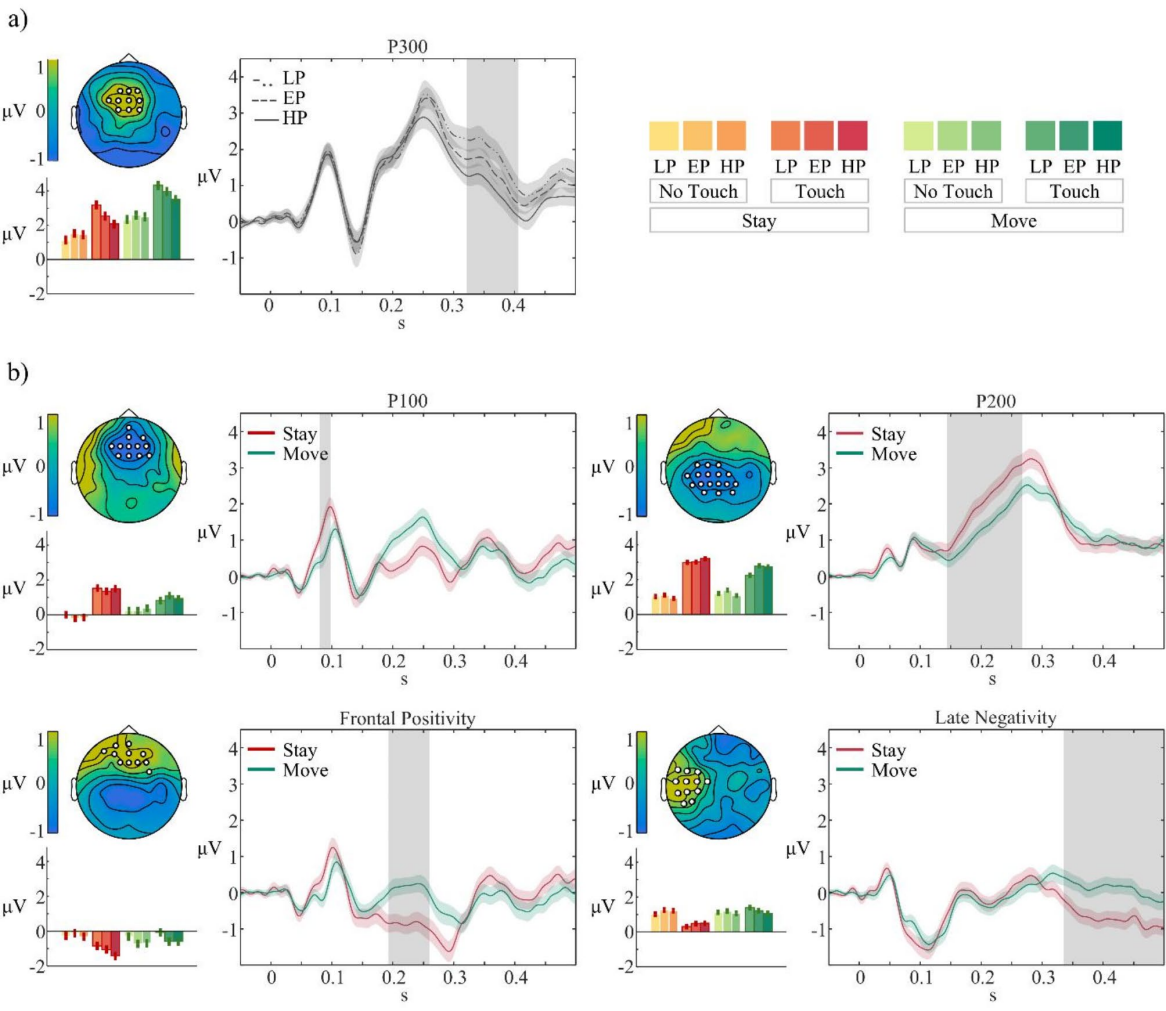
Electrophysiological results. (**a**) Interaction effect stimulation x probability; (**b**) Interaction effect stimulation x movement. All panels show the ERP plot of the subtraction of each *touch* condition to the corresponding movement specific average of *no-touch* conditions and then averaged per condition (averaged across movement types for panel a; averaged across probability conditions for panel b). The average of the electrodes comprising the cluster are plotted. Gray shaded areas represent significant time points with *p*_FWE_ < 0.05 and line contours are standard errors. Scalp distributions represent the difference between ERP plots across the significant time window. Bar-plots show the values of each condition across the significant time window, with standard errors. Legend on the upper right side of the image refers to all bar-plots in the panels. For a depiction of the ERPs before subtraction, please refer to Figure S2. LP, EP, HP = *low*, *equal*, *high* probability, respectively.

To test for differences across electrophysiological components evoked by the electrical pulse that are explained by self- or external- generation, while adjusting for the effects of a control *no-touch* condition and the effect of stimulus predictability, we investigated the interaction stimulation x movement, which revealed 4 main clusters reaching significance (see Fig. 4a). The earliest significant difference that survived cluster-level FWE correction in the potentials evoked by the electrical stimulation, minus of the corresponding *no-touch* control condition, was a positive deflection observed in frontal electrodes, starting at 0.080 s and ending at 0.098 s post stimulus (peak at 90 ms, centroid: F2, pFWE = 0.029, F = 20.76). The positive potential (P100) generated as the consequence of a movement was reduced (less positive) compared to the corresponding potential generated while staying still. Later in time, the analyses revealed two clusters surviving cluster-level FWE correction in frontal centro-parietal electrodes (peak: 160 ms, centroid: C1, pFWE = < 0.001, F = 21.04) and centro-frontal regions (peak = 244 ms, centroid = AF4, pFWE = 0.001, F = 18.02) respectively. Within the first cluster, the potential evoked by self-generated electrical stimulations was reduced in amplitude compared to the corresponding response in passive trials. In the second cluster, the results indicated an enhancement effect, such that in centro-frontal electrodes the average SEP showed a greater voltage during *move* trials. At first look, it might seem that the two potentials share similar substrates, however, a detailed analysis of the scalp topographies and the centroid of the respective clusters (see supplementary Figure S1) indicates that the difference at around 200 ms between self- and other-generated stimulations is greater in a centro-lateral electrode (C1) and then extends in time to central parietal bilateral sites. We will refer to this component as the P200, mostly because the centroid peaks much earlier in time (160 ms), compared to the effect observed at frontal sites (244 ms). Lastly, our results pointed out a cluster within parietal contralateral electrodes (peak: 395 ms, centroid: C3, pFWE < 0.001, F = 26.89) in which actively generated stimuli generated a suppressed negative potential compared to passively attended ones.

To test for differences between probability conditions, while controlling for the effects of the respective *no-touch* condition and of movement conditions, we investigated the interaction of stimulation x probability, that revealed one main cluster that reached significance at FWE correction for multiple comparison, located at centro-frontal electrodes over a time window between 0.322 and 0.406s after stimulus onset. This cluster will be referred to as the P300 (centroid: FCz, *p*_FWE_ = 0.012, *F* = 13.68). The P300 showed a parametric modulation, in the way that low probability conditions exerted a greater amplitude, while the *high* probability condition resulted in a suppression of the waveform (see Fig. 4b).

The interaction stimulation x probability x movement resulted in no significant clusters.

## Discussion

The investigation of sensory attenuation requires a multitude of control of concurring factors that could otherwise explain the attenuation normally observed between self- and externally-generated stimuli. With the present experiment we validated a novel VR paradigm that allowed for a more comprehensive control of stimulus properties, attentional requirements and other predictability factors during a modified probabilistic learning task. The electrophysiological results show that early- (P100) and mid-latency (P200) ERP components are suppressed for self- compared to externally-generated stimulations, which is a well-known finding of sensory attenuation. Moreover, self-generated stimuli elicited an enhancement of a later component in fronto-central sites, as well as a suppression of a later waveform at contralateral electrodes. The contrasting nature of these effects to stimulus predictability indicates that the phenomenon of sensory attenuation prevailed when controlling for other factors. Stimulus predictability was instead associated with a later attenuation effect for *high*-probability stimuli of the fronto-central P300.

*Sensory attenuation and possible confounds* Unlike previous studies investigating sensory attenuation, because we used VR to present somatosensory stimuli with different probabilities, we could obtain control *move* and *stay* trials that did not result in any electrical stimulation. This was important because the pre-stimulus waveform differed substantially across movement conditions. By specifically investigating the interaction term between movement and stimulation type, we could directly compare the evoked effect of touch in the different movement and probability conditions while controlling for the effects induced by the generation of a motor plan (*move* condition) or by passively waiting for a stimulation to occur (*stay* condition). Please note that the control conditions could also drive the interaction effects (see Figure S2), which is expected and relies on the assumption that there is no interaction between each movement and stimulation condition (i.e., the electrophysiological activity recorded in *move* conditions were identical across touch conditions), which is likely the case in our data as we observed no differences in the pre-stimulus period within *move* and *stay* trials, i.e., the difference between *touch* and *no-touch* trials comes down to zero before stimulus.

Moreover, this approach also avoids the possible problem that directly subtracting a control condition from a condition of interest might induce a spurious difference driven by the control condition itself^30^. Thus, we can assume that the present analyses allowed to directly compare the potentials evoked by a stimulus when self- or externally-generated (minus a control *no-touch move* or *stay* condition) across different stimulus predictability conditions.

Concerning the electrophysiological data, results revealed a suppression for self-generated stimuli at around 100 ms post-stimulus (P100) and at around 200 ms (P200). Components within the same time window have been associated with conscious perception^101–103^ and, more relevant to the scope of this paper, have also been demonstrated to be modulated by stimulus intensity^104–107^, attentional requirements^52–58^ and, despite evidence in the somatosensory modality is scarce, also by stimulus predictability in time^19,61–63^. These factors are also thought to impair the investigation of sensory attenuation as they might conflate the electrophysiological correlates of self-generated stimulations when left uncontrolled^37,50,60^. In trying to account for these aspects, we developed a VR setup that, similar to earlier studies in sensory attenuation in virtual environments, was designed to enable participants to perform naturalistic arm movement, reach and interact with virtual objects^76,79^, while controlling for the intensity and timing of tactile stimuli presented to participants^77,78^.

As in previous VR setups^75^, stimuli were directly triggered by touching the virtual object and were independent from the velocity at which the ball approached (or got approached by) participants’ index finger. In this way, we could account for the additional mechanical stimulation that would otherwise be present when pressing a button to generate a stimulation^37,51^. Attentional resources were also controlled across conditions, not only because immersive VR settings might lead to higher levels of concentration when performing a task^108,109^ but also, given that active hand and virtual ball movements occurred to and from both sides, we ensured spatial attention to be equal across conditions. More importantly, attentional resources were balanced across *stay* and *move* trials. Like previous studies that required participants to undergo interfering tasks such as counting the number of stimulations^50,110^ or estimating the time interval between consecutive stimulations^111^, in the present study volunteers needed to attend stimuli independently of whether they were presented in the *move* or *stay* conditions to form some expectations about their appearance. On a similar point, it is well known that instructing participants to refrain from performing eye movements during moving object fixation have been demonstrated to affect both early and late evoked EEG responses^112^. On the broader cognitive level, it is possible that the perception of the somatosensory stimuli was not optimal because participants were not fixating the exact point where the ball was being touched by the virtual finger. On one hand, it is unlikely that both electrophysiological and cognitive effects of restraining from eye movements would have impacted sensibly our data as the whole virtual scene was within the field of view of the headset; on the other hand, this effect, if present, was likely constant throughout conditions, thus it would have likely not affected the present results.

Volunteers could always predict the moment in time in which the ball would have administered (or not) the electrical pulse, not only in *move* trials as they were performing goal-directed actions, but also in *stay* trials because they could see the virtual ball approaching their index finger. Similar controls over stimulus temporal predictability have been adopted before^50^. Ball velocity was adjusted to each participants’ personal pace, as measured during the training phase. Response times comparison across conditions indicated that participants moved slower in *move* trials than the ball during *stay* trials. As a possible explanation, it is unlikely that participants kept the same movement pace from the training phase throughout the experiment, leading to an underestimation of the personalised response times during the training phase. However, it is unlikely that this difference affected the electrophysiological results. If the different trial lengths had led to a difference between the electrophysiological correlates at any point of the *stay* and *move* conditions, it would have affected both the *touch* and the respective control *no-touch* trials similarly, and therefore it should cancel out.

Attenuation of components at around 100 ms post stimulus is a common result obtained by electrophysiological studies investigating sensory attenuation in the auditory^16,41,49,50,113^, visual^44–46,114,115^ and somatosensory domain^47,48^. Similarly, suppression of P200 has been often reported in electroencephalography studies that investigated sensory attenuation either in the auditory^41^, visual^44,46^ and somatosensory modality^48^. By replicating the electrophysiological suppression for self-generated movement, we believe to also validate the present novel VR paradigm, which adds on the scarce literature of existing VR studies that investigated this phenomenon via EEG^79^. Differently from previous evidence, however, our setup also holds the advantage of being able to control for a series of confounding factors, not only replicating previous electrophysiological findings of sensory attenuation but also substantiating the validity of this phenomenon.

Assuming that sensory attenuation is not better explained by other concurring factors, it is likely that the electrophysiological attenuation for self-generated stimulations represents a reduction in the perceived intensity of the consequences of one’s own movements^18^. Corroborating this hypothesis, previous behavioural studies reported a reduced sensitivity^22,116^ or intensity^20,21^ in the perception of self-generated stimuli. Furthermore, previous results have demonstrated a direct link between subjective intensity reports and the somatosensory N140 and P200^48^ and the visual P2^46^, thus substantiating the idea that suppression of early- and mid- latency ERPs is accompanied by a decrease in subjective intensity. Although we did not collect stimulus intensity ratings and, thus, we could not directly test these findings, it is plausible that our results reflect the same subjective attenuation.

In trying to disentangle the underlying mechanisms of sensory attenuation, we did not only exert a comprehensive control over several intervening factors, but we also modulated the expectation of receiving a stimulation across conditions, regardless of whether it was self- or externally-generated. Previous studies that formally investigated this problem required participants to undergo sequences of highly predictable stimulation identities (constant or more predictable pitch) and totally unpredictable stimulations (variable pitch)^28,49^ or by contrasting stimuli that were congruent or incongruent to previous learned action-effect contingencies^22,72^. Evidence indicates that sensory attenuation is not better explained by stimulus predictions, either according to electrophysiological measures^28,49^ or subjective intensity rates^22,72^. Our results indicate that self-generated stimulations exert a greater electrophysiological suppression (in the P100 and P200) compared to externally administered stimuli, independent of the expectation of stimulus administration. Therefore, sensory attenuation can’t be better explained by stimulation predictability and might indicate that this phenomenon is not based upon unspecific prediction mechanisms, but that it is rather driven by specific motor predictions that are put forward when performing an action^35,117,118^.

*Further electrophysiological effects of self- vs. externally produced stimuli* Contrary to our expectation, however, later in time our results also pointed out another component at centro-frontal electrodes that showed an enhancement for self-produced compared to externally produced stimuli. Similar enhancements of centro-frontal positivities for self-generated stimuli, although sparse, have been described previously. Bednark and colleagues^49^ reported that the P3b was enhanced for self- compared to externally-produced stimuli, with a greater increase for stimulations that were incongruent to predictions. The authors attribute this enhancement effect to a possible link between the context updating hypothesis of the auditory P3 and the comparator model underlying sensory attenuation. More specifically, they hypothesise that in both mechanisms a stimulation is compared against an internal model which generates a prediction about said stimulation (either motor-specific and not); it is possible therefore that the anticipation of a stimulation is codetermined by both motor and non-motor predictions (see also Waszak & Herwig, 2007). Similar claims have also been advanced by Harrison and colleagues^50^, who speculated the existence of an “additive” effect of different predictive information. This claim might be substantiated by evidence of enhanced stimulus processing due to movement^120,121^. Although speculative, our results of enhanced fronto-central positivities may align with these explanations.

Lastly, another component that showed a modulation for self- compared to externally-generated stimulations was a negativity at parietal contralateral electrodes. Some findings suggest that similar potentials are related to somatosensory information maintenance in working memory^122–124^. Although participants were not actively required to rehearse any information during the task, it is plausible that they maintained some representations of the stimuli in memory to execute the learning task. Although, the reported suppression of the later potential for self- compared to externally generated stimuli might also represent a facilitation of stimulus processing specific to actively generated stimuli^120,121^. To our knowledge, the present study is the first to report similar effects in late-latency components for self- compared to externally-generated stimulations and its interpretations are to be considered exclusively as speculative, especially considering that similar potentials are known to be affected by methodological issues^125^.

*Electrophysiological effects of stimulus probability* Concerning the effect of stimulus expectation, our analyses revealed another centro-frontal cluster (P300) that showed a parametric effect over probability conditions (greater voltages for low-probability stimulations), independently whether participants actively produced the stimulation or passively perceived it. In the literature, the P300 has been oftentimes associated to the detection of deviant stimuli^126–129^ or, more generally, stimulus novelty or saliency^126,130–132^ and it seems to be involved in more endogenous processes, such as post-perceptual stimulus evaluation^133–136^. In line with this evidence, our results indicated that the stimulation drove a stronger effect when it was more unexpected (low probability conditions), suggesting that stimulus probability still affects stimulus perception (independently of the movement type) but in a much later time window.

Lastly, our paradigm could also be framed in the context of active sensing^10–12^, i.e., participants were actively controlling their sensory system to parse sensory information. In contrast to exploratory tasks, commonly used in the framework of active sensing^137,138^, the movement our subjects performed was limited to only two spatial positions. The probabilistic nature of the electrical pulses, when probing a virtual ball, could still be interpreted as information gathering and its effect on the P3 as a plausible consequence of the updating of the cognitive model by sensory information^10^. Nonetheless, further research is required to precisely frame sensory attenuation and its underlying mechanics in the active sensing framework^139,140^.

## Conclusions

Through our novel VR paradigm, we accounted for differences between self- and externally-produced stimuli in an ecological setup while controlling for other explaining factors that could impair the investigation of sensory attenuation, such as stimulus properties, attentional resources and stimulus predictability in time. Additionally, the setup was implemented to modulate stimulus expectation across conditions. Our results revealed an attenuation effect for self-generated stimulations in the P100, P200, and late-latency SEPs, as well as an enhancement of mid-latency potentials. These effects were independent from stimulus predictability, which instead modulated the P300 component. Therefore, we can conclude that the attenuation effect observed in the present experiment is not better explained by other explaining factors and is resilient to predictability manipulations, suggesting that sensory attenuation is a standalone phenomenon. The resilience of this phenomenon supports the concept that action indeed is a fundamental aspect of how we perceive ourselves and the world around us.

## Electronic supplementary material

Below is the link to the electronic supplementary material.

Supplementary Material 1.

## Data Availability

The data can be provided on request, taking into account the data protection guidelines.The corresponding scripts for data analysis and for replicating figures are available here: https://github.com/Neurocomputation-and-Neuroimaging-Unit/SensAtt_Pred.
