# Supplementary material for: Investigation of sensory attenuation in the somatosensory domain using EEG in a novel virtual reality paradigm
Source: Sci Rep. 2025 Jan 22;15:2819. doi: 10.1038/s41598-025-87244-9 (PMC11754869; doi:10.1038/s41598-025-87244-9)
Supplement: Supplementary file 1 — Supplementary Material 1. [file 41598_2025_87244_MOESM1_ESM.pdf]

Investigation of sensory attenuation in the somatosensory domain using EEG in a novel virtual reality paradigm

Giannini, G. [a, b, *], Nierhaus, T. [a], Blankenburg, F. [a, b]

[a] Neurocomputation and Neuroimaging Unit (NNU), Freie Universität Berlin, Berlin, Germany
[b] Berlin School of Mind and Brain, Humboldt Universität zu Berlin, Berlin, Germany
* Corresponding author. email: gianluigi.giannini@fu-berlin.de

Supplementary Information

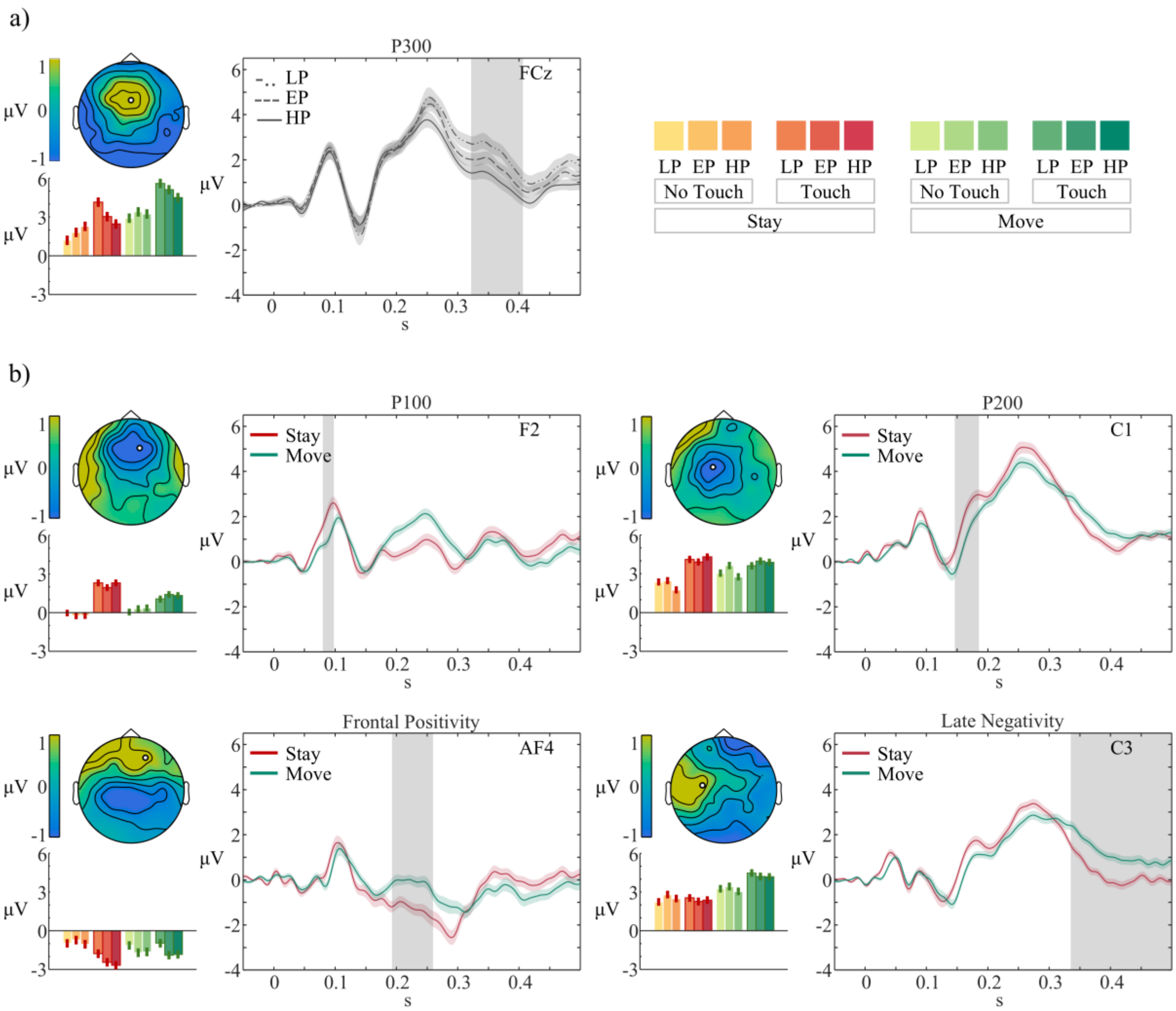

Figure S1: Electrophysiological results – peak channel (centroid) plots. (a) interaction effect stimulation *x* probability; (b) interaction effect stimulation *x* movement. All panels show the ERP plot of the subtraction of each *touch* condition to the corresponding movement specific average of *no-touch* conditions and then averaged per condition (averaged across movement types for panel a; averaged across probability conditions for panel b). Only the peak electrode of the cluster is plotted and the respective label is available in the right upper corner of the plot. Gray shaded areas represent significant time points with $p_{FWE} < 0.05$ and line contours are standard errors. Scalp topographies represent the difference between ERP plots across the significant time window. Bar-plots show the values of each condition across the significant time window, with standard errors. Legend on the upper right side of the image refers to all bar-plots in the panels. LP, EP, HP = *low*, *equal*, *high* probability, respectively.

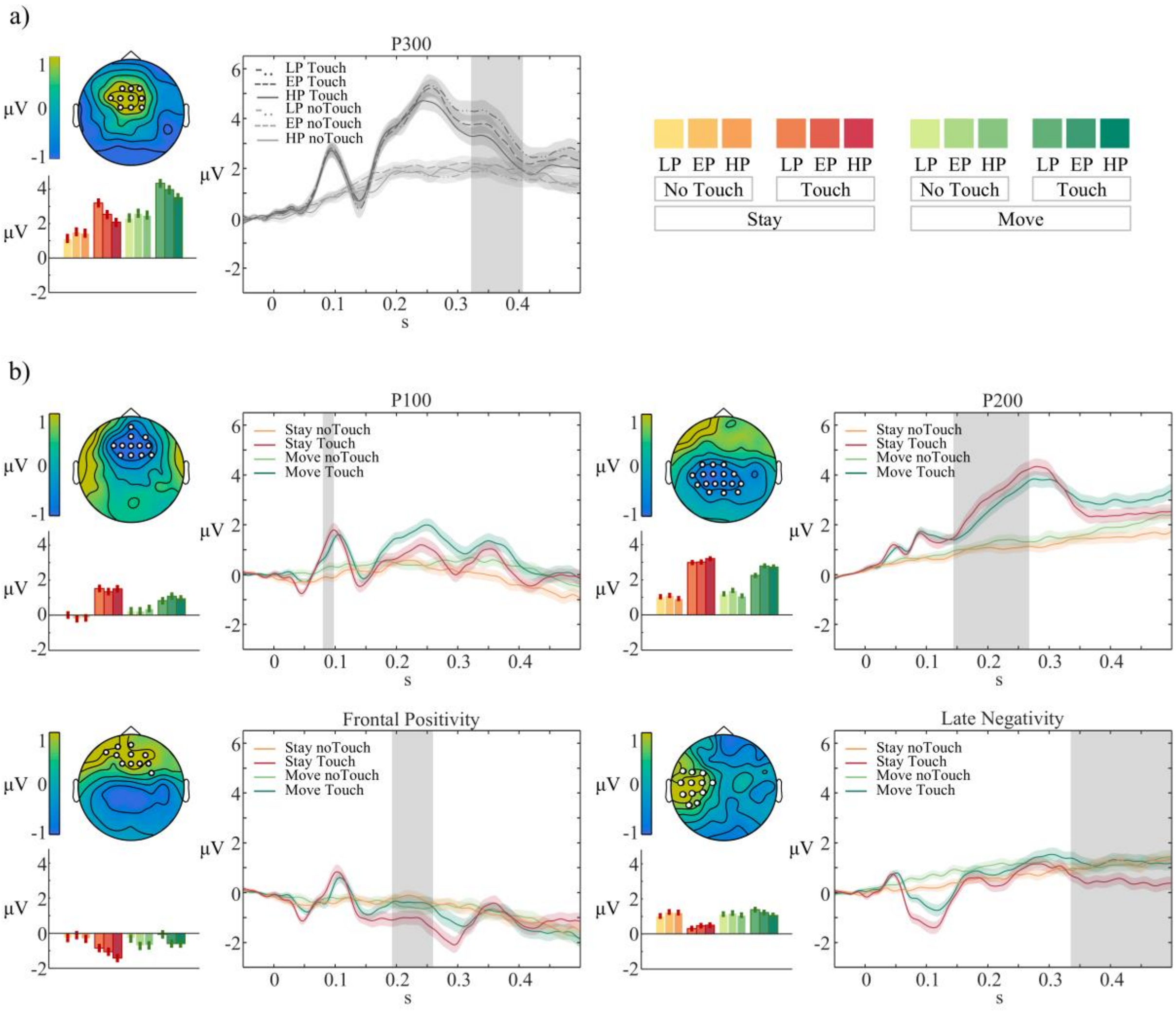

Figure S2: Electrohysiological results – non-subtracted ERPs. (a) interaction effect *stimulation x* probability; (b) interaction effect stimulation *x* movement. All panels show the ERP plot before subtraction of each *touch* condition and the corresponding *no-touch* conditions (averaged across movement types for panel a; averaged across probability conditions for panel b). The average of the electrodes comprising the cluster are plotted. Gray shaded areas represent significant time points with $p_{FWE} < 0.05$ and line contours are standard errors. Scalp distributions represent the difference between ERP plots across the significant time window. Bar-plots show the values of each condition across the significant time window, with standard errors. Legend on the upper right side of the image refers to all bar-plots in the panels. Please note that some interaction effects are driven by difference in control *no-touch* conditions rather than *touch* conditions. This is plausible under the assumption that the same electrophysiological activity associated to movement (or staying still) was equally present in *touch* and *no-touch* conditions. LP, EP, HP = *low*, *equal*, *high* probability, respectively.
